# Cricoarytenoid joint: histological changes during aging

**DOI:** 10.1590/S1516-31802001000200011

**Published:** 2001-03-02

**Authors:** Rogério Aparecido Dedivitis, Márcio Abrahão, Manoel de Jesus Simões, Osvaldo Alves Mora, Onivaldo Cervantes

## INTRODUCTION

Voice changes related to the aging process have been attributed to irregularities in vocal fold vibration, glottal incompetence, vocal fold atrophy, and laryngeal tension. Associated with these mechanical factors are the presence of systemic illnesses and the normal age-related growth and histological changes noted in the laryngeal connective tissues.^1^ The effect of physiological aging on the structure and function of the cricoarytenoid joint (CAJ) is not well known. The presence of calcium deposits seen on roentgenogram^2^ and muscle degenerative changes^3^ are thought to be indicators of CAJ aging. The CAJ is a diarthrosis formed by the multiaxial apposition of the articular facets of the arytenoid and cricoid cartilages.^4^ The ossification pattern is typically endochondral in the laryngeal cartilages with little perichondrium participation.^5^ A true ossification process starts in the laryngeal cartilages as early as the twentieth year. The cartilage of the larynx that ossifies is of the hyaline variety. Fatty and hematopoietic marrow are observed after the thirties on magnetic resonance imaging.^6^

## METHODS

For this study, fresh human CAJ removed from twenty-four postmortem examinations was evaluated. The specimens were obtained from the Forensic Medicine Office of the Civil Police in Santos, State of São Paulo, from November 1999 to January 2000. The cadavers were Caucasian and male and they were divided into three groups with eight specimens each: group I or adolescents – from 15 to 20-years-old; group II or adult – from 25 to 35; and group III or elderly – from 60 to 75. Smoking and drinking histories were not available. None of the cadavers had a laryngeal cause of death. The specimens were preserved in 10% neutral buffered formalin and underwent decalcification. They were stained with hematoxylin-eosin, Masson trichrome, picro-sirius and Weigert elastic stain. The macroscopic aspect was evaluated according to the color and the consistence. Ossification and bone marrow were analyzed according to their presence or absence. A measurement eyepiece coupled to a light microscope was used for perichondrium thickness calculation. Four measurements were taken for each specimen. The data obtained were represented as averages and standard deviations. The averages were compared according to the variance analysis method with significance of 95%. When the averages for one group were different from the others, Bonferroni's method was used to detect them. Fisher's Exact Test was used to verify the difference between the frequencies of the groups, with significance of 95%.

**Figure:**
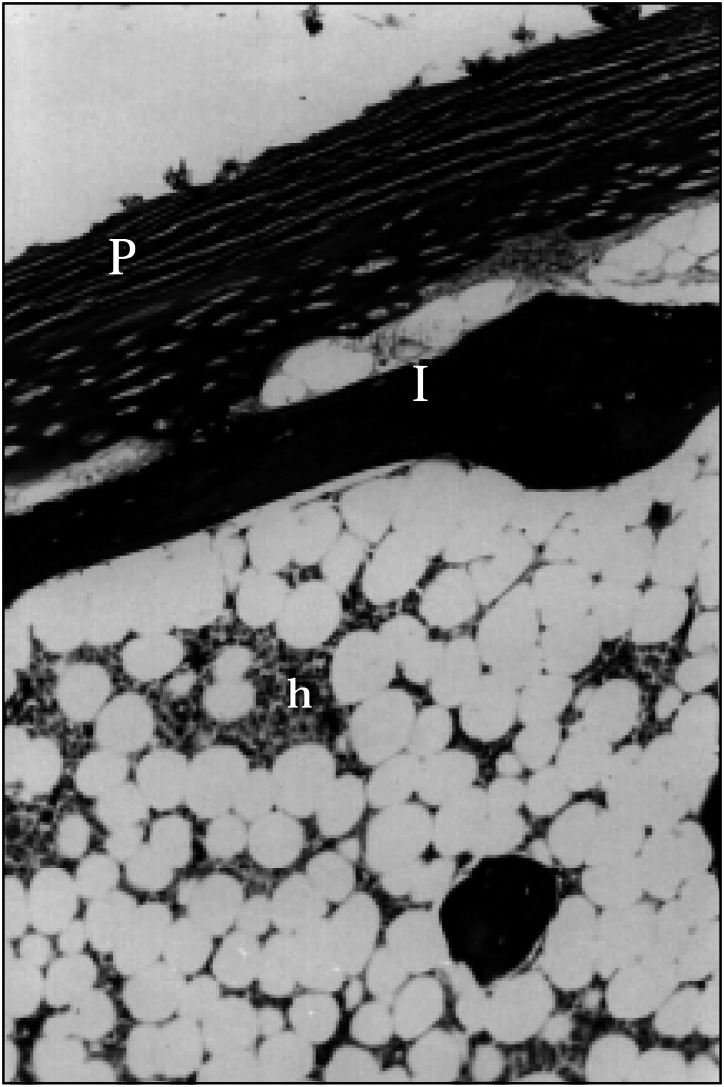
Photomicrograph of an elderly CAJ with typical bone marrow surrounded by lamellar bone tissue (l). Hematopoietic tissue (h) is present. The perichondrium is thick. (trichrome stain, x140).

## RESULTS

The adolescent and adult cartilaginous specimens were white nacreous, homogeneous, smooth, shiny, fibrous elastic and surrounded by typical muscle fibers. In the elderly group, the cartilaginous specimens were yellow, heterogeneous, rugose, with no brightness and hard, with the muscle tissue also being hard. The central portion of six specimens presented an appearance typical of bone marrow.

In group I, the cartilages were made up of typical hyaline cartilage, with thin perichondrium consisting of chondroblasts, mesenchymal cells and collagen fibers. In group II, while four articular specimens were of typical hyaline cartilage, the other four specimens showed ossification in delimited areas in the deeper parts of the cartilages. The bone tissue was lamellar. In two cases, the bone cavities were full of adipose tissue. The perichondrium had an appearance similar to that of the adolescent group. In group III, most of the hyaline cartilage was replaced by typical lamellar osseous tissue with the presence of some Haversian systems.

Hyaline cartilage tissue surrounds the bone as a thin layer with typical appearance. In the deep portion of these specimens there was a bone cavity with true hematopoietic tissue in six and adipose tissue in another two (P<0.05). The perichondrium was thicker and had a lot of collagen fibers. Elastic fibers were present in the outer part of the perichondrium in small quantity in the different groups. Perichondrium thickness (mm) was for the respective age groups: 0.2712 (SD 0.041); 0.3025 (SD 0.052); and 0.4625 (SD 0.037).

## DISCUSSION

There is little known about how changes in CAJ due to histological aging correlate with alterations in perceptive and acoustic changes. The CAJ, which is a very precise joint, shows the impact of age in a loosening of the capsule and in the erosion of its surfaces. As a result, the meticulous approximation of the arytenoids, which is indispensable for the emission of a given tone, is jeopardized^7^. Voice changes related to the aging process have been attributed to irregularities in vocal fold vibration, glottal incompetence, vocal fold atrophy, and laryngeal tension.^1^ Regressive processes in the articular cartilages such as degeneration of surrounding muscle fibers, and progressive processes such as ossification, are continuous and occur mainly in the elderly.^8^ The ossification occurs after the forties and is caused by an involution of the cartilaginous tissue and its replacement by typical lamellar bone with true hematopoietic tissue. The presence of hematopoietic tissue in ossified cartilages of the elderly was a significant finding that is in accordance with other reports.^6,9^ It was found even among the adult group.

## CONCLUSION

We conclude that: (1) in spite of its absence in adolescence, ossification occurs in cricoid and arytenoid cartilages from the onset of adulthood, becoming more intense in the elderly group; (2) bone marrow is formed in CAJ ossification tissue with hematopoietic tissue in group III; (3) the perichondrium becomes thicker in both cartilages among the elderly; and (4) the quantity of elastic fibers is small, independent of the age group, and they are seen in the perichondrium.
